# The Effect of Temperature on the Development of *Trilocha varians* (Lepidoptera: Bombycidae) and Control of the Ficus Plant Pest

**DOI:** 10.21315/tlsr2019.30.1.2

**Published:** 2019-01-31

**Authors:** Norasmah Basari, Nurul Salsabila Mustafa, Nur Elya Nabila Yusrihan, Chin Wei Yean, Zainal Ibrahim

**Affiliations:** 1 School of Marine and Environmental Sciences, Universiti Malaysia Terengganu, 21030 Kuala Nerus, Terengganu, Malaysia; 2 Majlis Bandaraya Kuala Terengganu, Menara Permint, Jalan Sultan Ismail, 21000 Kuala Terengganu, Terengganu, Malaysia

**Keywords:** Moth, Ornamental Plants, Malathion, Fipronil, *Ficus* spp., Tropical Environment, Kupu-kupu, Pokok Hiasan, Malathion, Fipronil, *Ficus* spp., Persekitaran Tropika

## Abstract

*Ficus* plants are commonly planted as ornamentals along roadsides in Malaysia. In 2010, *Ficus* plants in Kuala Terengganu were found to be attacked by a moth, identified as *Trilocha varians*. The larvae of this moth fed on *Ficus* leaves causing up to 100% defoliation. This study was conducted to determine the life cycle of *T. varians* under two different environmental temperatures and to control this pest using two different insecticides. Our findings showed that there were significant differences in the time taken for eggs to hatch and larval and pupation period between low and high environmental temperatures. Results also showed that fipronil had lower LT_50_ and LT_95_ than malathion. This study provides new information on the life history of *T. varians* under two different conditions and the efficiency in controlling *T. varians* larvae using insecticides. The results of this study are important for future management in controlling *T. varians* population especially in Kuala Terengganu, Malaysia.

## Abstract (ms)

Pokok *Ficus* biasa ditanam sebagai tanaman hiasan di sepanjang jalan raya di Malaysia. Pada tahun 2010, pokok *Ficus* di Kuala Terengganu didapati telah diserang oleh kupu-kupu yang dikenali sebagai *Trilocha varians*. Larva kupu-kupu pemakan daun ini boleh menyebabkan pokok *Ficus* mengalami kehilangan daun sehingga 100%. Kajian ini dijalankan untuk menentukan kitar hidup *T. varians* di bawah dua suhu persekitaran yang berbeza dan untuk mengawal serangga perosak ini menggunakan dua jenis insektisid. Keputusan menunjukkan terdapat perbezaan yang signifikan bagi masa yang diambil untuk telur menetas dan jangka masa bagi larva dan pupa di antara suhu persekitaran rendah dan tinggi. Keputusan juga menunjukkan fipronil mempunyai LT_50_ dan LT_95_ yang rendah berbanding malathion. Kajian ini memberi maklumat baharu berkaitan kitar hidup *T. varians* di bawah dua keadaan berbeza serta kecekapan kawalan larva *T. varians* menggunakan insektisid. Keputusan kajian ini penting untuk pengurusan kawalan populasi *T. varians* pada masa hadapan terutamanya di Kuala Terengganu, Malaysia.

## INTRODUCTION

*Ficus* spp. such as *Ficus benjamina* L. are commonly planted as ornamental plants in many areas in Malaysia. *Ficus* plants can adapt well in high temperature environment. The plants are easy to trim in various shapes to enhance aeasthetic aspects especially in the cities. However, *Ficus* plants are not susceptible to pests. There are reports about the infestations of pest insects on *Ficus* plants especially by moth larvae in other countries such as the Philipines (Navasero *et al*. 2013) and India (Kedar *et al*. 2014; Singh & Brar 2016).

In Bandaraya Kuala Terengganu, *Ficus* plants had been found to be infested by a moth, identified as *Trilocha varians* (Walker 1855) of Family Bombycidae since 2010. The infestation of *T. varians*, mainly by the larvae (caterpillars), causes up to 100% defoliation to the trees. This severe defoliation eventually kills the plants and degrades the aesthetic value of the city.

Control of *T. varians* in the field was conducted regularly by pest controllers using malathion. However, the problem of infestations still persisted until 2016. Hence, treatment with another type of insecticide, fipronil, was introduced in 2016. Since then, numbers of infested trees were reduced. However, no empirical study was conducted to determine the effectiveness of malathion and fipronil to control the pest of *Ficus* plants in Malaysia. There was also no study conducted on the life history of *T. varians* in the Malaysian environment. Hence, this research was conducted to determine the life cycle of *T. varians* and to control the pest in Kuala Terengganu, located in the east coast of Peninsular Malaysia. The life cycle of *T. varians* was recorded under two conditions; controlled and uncontrolled conditions. This was done because the temperature in the east coast of Peninsular Malaysia is mainly affected by the monsoon season. In general, Malaysia experiences uniform temperature throughout the year but during the monsoon season (rainy season) in the east coast of Peninsular Malaysia, from October until March, environmental temperature will be a bit lower, ranging between 23°C to 26°C while normal temperature (dry season, from April until September) ranges between 27°C to 32°C. The aims of this study are to determine the life history of *T. varians* under two different temperature conditions and to determine the efficiency of using malathion and fipronil to control the population of *T. varians*.

## MATERIALS AND METHODS

### Sampling Site

Samples were collected directly from infested *Ficus* plants along Jalan Kemajuan in Bandaraya Kuala Terengganu (05°19′16.7628″N, 103°08′10.2264″E), Terengganu.

#### 1. Life history of *T. varians*

**Figure 1:**
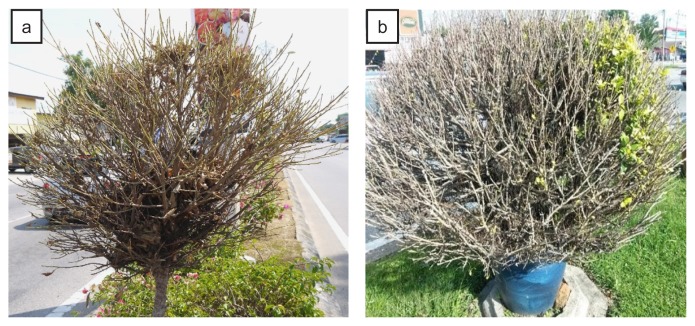
(a) Total (100%) defoliation and (b) about 90–95% defoliation of *Ficus benjamina* trees by the larvae of *T. varians* in Bandaraya Kuala Terengganu.

Samples of various stages of *T. varians* larvae were collected manually using soft forcep from the infested trees (Fig. 1). All samples were kept in transparent plastic containers and brought to the laboratory in Universiti Malaysia Terengganu (UMT) for rearing and monitoring.

In the laboratory, at least three larvae were placed in 500 ml transparent plastic pet containers. Larvae were fed with fresh *Ficus benjamina* leaves daily until they reached pupal stage. After adult emergence, a pair of the moth (male and female) were placed in a jar for mating purposes. After the mating period, leaves in each containers were examined for the presence of eggs. Leaves with eggs were put in different containers and the development of the larvae were recorded daily under two different conditions; controlled (24°C ± 0.6) and uncontrolled (30°C ± 2.0) temperature conditions. Once the eggs hatched, larvae were separated into different containers equipped with *F. benjamina* leaves for food. Developmental progress was recorded daily. During the developmental process, new *F. benjamina* leaves were provided daily for the larvae until they became pupae. If the larvae or pupae died before reaching adult stage the data were removed. In total, 20 individuals were successfully reared under controlled condition and 16 individuals under uncontrolled condition.

#### 2. Control of *T. varians* using insecticides

Two types of insecticides were used to test for control efficiency against *T. varians* larvae, namely malathion (57% active ingredient) and fipronil (5% active ingredient). Twenty *Ficus benjamina* seedlings (*Ficus* plants) in polybags were brought from nursery to the research area in the UMT campus. All *Ficus* plants used in this study had heights which ranged between 50 to 70 cm. All Ficus plants were divided into four groups: 1) Negative control, 2) Positive control, 3) Malathion treatment, and 4) Fipronil treatment.

Each group consists of five *Ficus* plants. Each *Ficus* plant in groups 2, 3 and 4 were infested with six larvae. Only larvae from stage five were used. The larval stages were determined based on the distinguished physical characteristics as mentioned by Navasero and Navasero (2014). In total, 30 larvae were used to infest *Ficus* plants in groups 2, 3 and 4 while no larvae were introduced to the *Ficus* plants under group 1 (negative control). Due to the size of the *Ficus* plants used in this study, only a small number of larvae were used to infest each *Ficus* plant. This was done to ensure leaves were still present when insecticides were sprayed onto the *Ficus* plants since larvae at stage five are highly active in terms of leaf consumption (Navasero & Navasero 2014). Experiment was conducted under uncontrolled environmental condition with temperature ranging between (28°C ± 2.0).

After the larvae were introduced to the *Ficus* plants, they were left for 24 h before the insecticides were sprayed. This was done to ensure all larvae were still alive before treatment with insecticides. The concentration of malathion and fipronil used in this experiment were the same as used by pest controllers during their monthly scheduled insecticide treatment in Kuala Terengganu (7.003 × 10^−3^ g/mol of malathion and 100 ml of 1.693 × 10^−4^ g/mol of fipronil, respectively). In this study, the different concentrations of both insecticides were used as these were the concentrations used by the pest controllers while treating the pest. Since we only focused on determining the pest mortality after insecticide applications, we did not test for the lethal concentration for both insecticides. After 24 h of larvae infestation, 100 ml of malathion was sprayed onto each *Ficus* plant in group 3 and 100 ml of fipronil insecticide was sprayed onto each *Ficus* plant in group 4. *Ficus* plants under groups 1 and 2 (negative and positive control) were sprayed with 100 ml of distilled water. All plants were covered with fine mesh net to prevent the larvae from escaping and also to prevent other pests from disturbing the experiment. The number of survival and mortality of *T. varians* larvae were recorded daily.

### Statistical Analysis

Difference in the developmental period of *T. varians* between controlled and uncontrolled conditions were analysed using Mann-Whitney test. For insecticide control efficiency, probit analysis was used to determine the lethal time 50 (LT_50_) and lethal time 95 (LT_95_). Both analyses were conducted using SPSS (v. 24).

## RESULTS

### Life History of *T. Varians*

**Figure 2:**
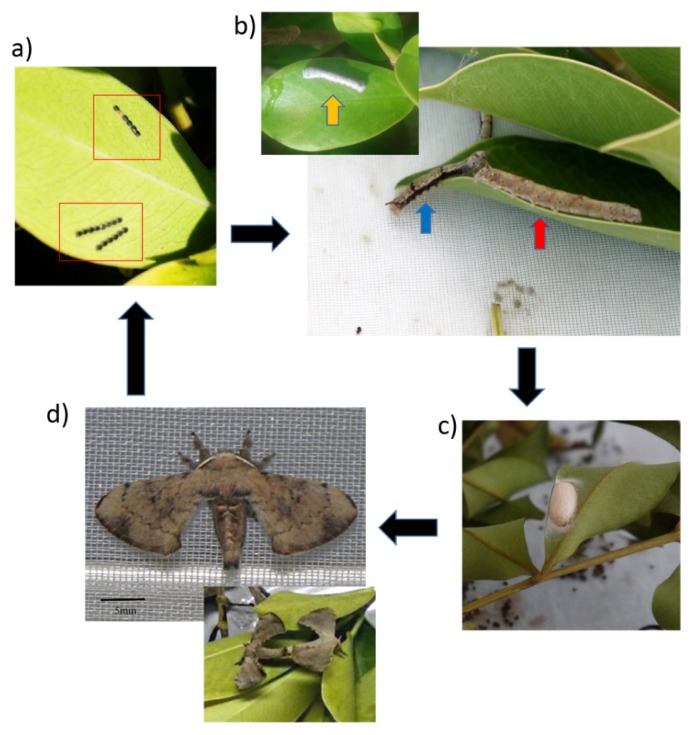
Life history of *T. varians*. (a) eggs mass (in red box), eggs became black in colour about 24 hours before hatch; (b) Yellow arrow: Third instar larvae covered in white powdery secretions, blue arrow: fourth instar larvae covered with loose powdery secretions, red arrow: fifth instar larvae without powdery secretions and brown-reddish in colour; (c) pupa in silk coccoon on *Ficus benjamina* leaf; (d) adult *T. varians* (lower picture shows a pair of *T. varians* during mating).

Under controlled condition (low temperature, 24°C ± 0.6), the eggs (Fig. 2a) took about five days (mean ± SE, 5.17 ± 0.38) to hatch and to become larvae. Larvae (Fig. 2b) stage lasted about 19 days (mean ± SE, 18.7 ± 1.59) before transformed into pupae (Fig. 2c). Pupae took six and a half days (mean ± SE, 6.55 ± 0.83) before emerging as adults (Fig. 2d). Under uncontrolled condition (high temperature, 30°C ± 2.0), eggs took a shorter time to hatch, only four and a half days (mean ± SE, 4.56 ± 0.89). They also spent a shorter time as larvae, about 15 days (mean ± SE, 14.94 ± 1.77) before transformed into pupal stage. Pupation period also took a shorter time, about five days (mean ± SE, 5.44 ± 1.03) before they emerged as adults. There were significant differences in the time taken between controlled and uncontrolled conditions for eggs to hatch (*U* = 73.5, *N* = 36, *p* = 0.008, *r* = 0.45), larvae period (*U* = 15, *N* = 36, *p* < 0.001, *r* = 0.77) and pupation period (*U* = 66, *N* = 36, *p* < 0.05, *r* = 0.53). Total developmental periods of of male and female *T. varians* were not significantly different under both controlled and uncontrolled conditions, (Controlled: male, 29.8 ± 2.04 days, female, 31 ± 1.33 days; *U* = 31.5, *N* = 20, *p* = 0.17, *r* = 0.31; Uncontrolled: male, 25 ± 0.94 days, female, 24.83 ± 2.22 days; *U* = 28.5, *N* = 16, *p* = 0.91, *r* = 0.03).

### Control of *T. varians* Using Insecticides

After treatment by malathion, 50% of mortality was recorded within 2.14 days (LT_50_ = 2.138) and 95% of mortality was recorded after 4.116 days (LT_95_ = 4.116) (Table 1). On the other hand, after treatment by fipronil, mortality of half of the population was recorded after 1.52 days and 95% of the population were found to be dead by 3.21 days (Table 1). All larvae (30 individuals) under positive control became pupae after seven days and emerged as adults after five days (on average).

## DISCUSSION

We conducted studies on the life history of *T. varians*, pest of *Ficus* trees in Kuala Terengganu, Malaysia under two environmental conditions and also conducted a test to determine the effectiveness of using malathion and fipronil to control the pest population.

Overall, the developmental period of *T. varians* from the egg stage to adult stage was longer under lower temperature (controlled condition) compared to under higher temperature (uncontrolled condition). Low temperature affected the development of the moth at an earlier stage, as suggested by Daimon *et al*. (2012), Lu *et al*. (2016) and Sibly *et al*. (2016). Hence, we suggest that the developmental period of *T. varians* could be longer during monsoon season from October until March. While during the dry season from April until September the developmental period was faster. This suggests that the defoliation of *Ficus* plant could be higher from October until March because of the abundance of larvae due to the delay in their development to become pupae. However, further experiments should be conducted to support this hypothesis. Life history of *T. varians* under controlled conditions in the current study is in line with a previous study conducted by Navasero and Navasero (2014). However, in this study we also conducted life history study under uncontrolled conditions that leads to a new finding where the life cycle was shortened under higher temperature.

To deal with the problem of defoliation of *Ficus* tree by the larvae of *T. varians* in Kuala Terengganu, two insecticides (malathion and fipronil) were chosen to determine the lethal time 50 (LT_50_) and lethal time 95 (LT_95_). Different concentrations were used in this study as these were the same concentrations used by pest controllers to control the pest in Kuala Terengganu. However, no study had been conducted before to determine mortality of this pest after being treated with the insecticides. Hence, we tested the lethal time using the same concentration used by pest controllers. Our results showed that both insecticides were suitable in controlling *T. varians* larvae. However, mortality of *T. varians* larvae caused by fipronil was faster than by malathion. The effect of fipronil on the LT_50_ was 1.519 days while malathion was 2.138 days. A previous study by Kumar *et al*. (2015) also found that fipronil had lower LC_50_ compared to malathion when testing foracaricide resistance in ticks infesting animals. The results of this study showed LC_50_ of fipronil was 1.68ppm and LC_50_ of malathion was 2764.2 ppm. This showed that fipronil was more toxic than malathion. Detailed studies looking at the lethal concentration of both insecticides will be conducted in the future.

Fipronil is a neurotoxic insecticide which belongs to phenylpyrazole group. Fipronil acts as a gamma-aminobutyric acid receptor (GABA) blocker in the insect body (Caboni *et al*. 2003). GABA functions as inhibitors of normal neural activity and prevents excessive stimulation of nerves. When fipronil is released into the nervous system, it will block the regular function of nerves and thus causes neural excitation, paralysis and death of the organism. Fipronil can kill insects by direct contact and ingestion. Fipronil has lower LT_50_ and LT_50_ towards larvae of *T. varians* because it is very effective against adult and larval stage of insect (Colliot *et al*. 1992; Zhang *et al*. 2015). Malathion is a insecticide which belongs to the organophosphates group. It is used outdoors to control many types of insect such as mosquitoes. It kills insects by preventing the normal function of nervous systems. Malathion binds to an enzyme that is released into the space between nerves causing continuous signal transfer between the nerves. Insects will become paralysed or are unable to breathe normally and die in the end. Malathion can be exposed by skin contact, ingestion and respiration. However, malathion can be tolerated by *Anopheles maculipennis*, a mosquito found in Northwest of Iran (Hasasan & Hossein 2010). This showed that malathion is less toxic and causes less harm to organism.

Overall, fipronil is a better insecticide to be used to control *T. varians* instead of malathion because it is more toxic and effective towards *T. varians* larvae even though the concentration used by pest controllers was lower campared to malathion (1.693 × 10^−4^ g/mol of fipronil vs. 7.003 × 10^−3^ g/mol of malathion). In terms of economical comparison, fipronil maybe more expensive compared to malathion. However, since the lethal time was shorter even at low concentration when using fipronil, it could help to minimise the uses of the insecticide and could reduce the number the pest rapidly. This will eventually reduce the number of plant loss due to severe infestation by the pest. Hence using fipronil could be more economical compared to malathion. There are many advantages in using chemicals to control pests. One of the major benefits is efficiency. The chemicals are manufactured to kill pests within the shortest time if appropriate concentration is applied. Chemical control also can be applied and is available anytime when a pest problem occurs. However, since certain pests could become resistant to certain insecticides after long exposure, it is suggested that different type of insecticides are used alternately to overcome this problem. Last but not least, to avoid effects on the non-target animals, the best precaution is to avoid applying insecticides under windy conditions to reduce the spread of the insecticide to the non-target areas.

## CONCLUSION

Diverse weather conditions can cause differences in the life cycle of *T. varians*. At a low temperature, the life cycle of this pest is longer but at a higher temperature the development of this pest is faster. Both insecticides, malathion and fipronil, were proven to be lethal to larvae of *T. varians*. However, our results showed that fipronil had low LT_50_ and LT_95_ than malathion. This means that fipronil is more effective to control *T. varians* larvae than malathion. This study provides basic information on the pest insects of *Ficus* plants and insecticides efficiency towards *T. varians* larvae in Kuala Terangganu. This study also provides new information on the life history of *T. varians* under two different conditions. The results of this study are important for future management in controlling *T. varians* population especially in Kuala Terengganu, Malaysia.

## Figures and Tables

**Table 1:** Control of *T. varians* using insecticides.

Insecticide	Probability	Estimate	Lower bound	Upper bound
Malathion	0.500	2.138	−1.292	3.244
	0.950	4.116	2.994	7.025
Fipronil	0.500	1.519	−0.633	2.648
	0.950	3.207	2.114	5.719
